# Medical Challenges in Caring for Older Adults With Type 2 Diabetes and Dementia: A Qualitative Interview Study Using SCAT to Compare Primary Care Physicians and Diabetes Specialists in Japan

**DOI:** 10.1002/jgf2.70154

**Published:** 2026-07-26

**Authors:** Shin‐ichi Taniguchi, Daisuke Son, Kazuoki Inoue, Shintaro Imaoka, Tsubasa Nakai, Yuma Otsuka, Shohei Taniguchi, Toshihiro Hamada, Naomi Sunami

**Affiliations:** ^1^ Department of Community‐Based Family Medicine, Faculty of Medicine Tottori University Yonago Japan; ^2^ Department of General Medicine Hino Hospital Association Hino Hospital Tottori Japan; ^3^ National Health Insurance Daisen Clinic Tottori Japan; ^4^ Department of Internal Medicine Nichinan Town National Health Insurance Nichinan Hospital Tottori Japan; ^5^ Department of Psychology, Faculty of Liberal Arts Teikyo University Hachioji Tokyo Japan

**Keywords:** dementia, diabetes specialists, elderly patients, primary care, qualitative study, type 2 diabetes

## Abstract

**Background:**

As populations age, older adults with type 2 diabetes and comorbid dementia are increasing. Dementia can impair diabetes self‐management and require physicians to reconsider treatment goals, family involvement, care coordination, and ethical decision‐making. However, few studies have explored physicians' specific challenges in such cases, particularly within the Japanese healthcare context.

**Methods:**

This qualitative study examined physicians' perceived difficulties in caring for older adults with type 2 diabetes and dementia. Semi‐structured interviews were conducted with six primary care physicians and six diabetes specialists in Japan. Transcripts were analyzed using the Steps for Coding and Theorization (SCAT) method to identify recurring themes and compare perspectives across physician groups.

**Results:**

Both groups reported challenges related to the gap between ideal and feasible care, dementia‐related disruption of self‐management, family involvement, emotional burden, and practical treatment adaptations. Primary care physicians more often emphasized preserving dignity, life context, longitudinal relationships, and coordination with other professionals. Diabetes specialists more often emphasized disease‐specific uncertainty, treatment‐goal adjustment, dementia diagnosis or disclosure, and pragmatic adaptations to maintain safety.

**Conclusions:**

Physicians caring for older adults with type 2 diabetes and dementia experience clinical, ethical, relational, and emotional challenges shaped by their roles and practice contexts. These findings highlight the need for educational and organizational support to help physicians negotiate individualized treatment goals, family involvement, and interprofessional coordination in diabetes–dementia care. Future research should evaluate whether such support can reduce physician burden and improve care quality.

## Graphical abstract

This study explores physicians' challenges in caring for older adults with type 2 diabetes and coexisting dementia. Based on qualitative interviews with primary care physicians and diabetes specialists in Japan, it identifies shared challenges and differently emphasized concerns across physician groups, including disruption of diabetes self‐management, family involvement, emotional uncertainty, treatment‐goal adjustment, and care coordination. The findings suggest the need for role‐sensitive educational and organizational support for physicians involved in diabetes–dementia care.

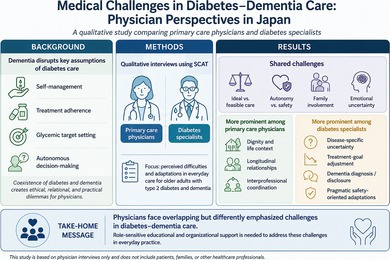

## 1 Introduction

The global prevalence of type 2 diabetes has risen dramatically in parallel with improvements in living standards and increasing life expectancy. According to the International Diabetes Federation, an estimated 10.8 million adults aged 20–79 years in Japan were living with diabetes in 2024, making diabetes a major public health issue in the context of population aging [1]. Type 2 diabetes is associated with both microvascular complications, such as retinopathy, neuropathy, and nephropathy, and macrovascular conditions like cerebrovascular disease and ischemic heart disease, placing a considerable burden on national healthcare systems.

As the aging population expands, a growing subset of older adults with diabetes are also developing dementia. Dementia is increasingly recognized as a common comorbidity among elderly diabetic patients, with studies reporting that individuals with diabetes have a significantly elevated risk of developing cognitive impairment compared to those without diabetes [2, 3, 4]. Once dementia is present, it can severely disrupt self‐care behaviors such as glucose monitoring, medication adherence, diet regulation, and exercise—essential pillars of diabetes management. Consequently, dementia alters the therapeutic goals and strategies for diabetes care, posing new ethical and emotional challenges for physicians.

Diabetes management inherently relies on patient self‐regulation. Patients are expected to make ongoing lifestyle adjustments in diet, physical activity, and medication adherence to maintain optimal glycemic control. Patient education, motivation enhancement, and individualized care plans are central components of effective diabetes treatment. In older adults, however, diabetes treatment must be individualized by considering cognitive function, functional status, frailty, multimorbidity, hypoglycemia risk, quality of life, and available social support [5]. The presence of dementia further complicates care due to memory loss and behavioral and psychological symptoms of dementia (BPSD), which may include agitation, aggression, delusions, or apathy [6, 7, 8, 9]. These symptoms not only hinder diabetes self‐management but also increase caregiver burden, healthcare costs, and the risk of early institutionalization [10, 11, 12].

While best practices in dementia care emphasize multidisciplinary collaboration, early diagnosis, appropriate use of cholinesterase inhibitors, and effective communication with families, studies suggest that such standards are often unmet in clinical settings [13, 14, 15, 16, 17]. Barriers include underdiagnosis, delayed recognition of symptoms, inadequate physician knowledge, and fragmented care systems [18, 19, 20]. For primary care physicians, providing continuous care to dementia patients often involves shifting roles—from treating disease to supporting caregivers and navigating complex psychosocial dynamics [21].

Recent qualitative research has also highlighted the difficulty of managing multimorbidity in patients with dementia, including challenges related to self‐management, consultation time, refusal of care support, care coordination, and fragmentation in free‐access healthcare systems [22]. However, diabetes–dementia comorbidity presents a particularly specific challenge because diabetes care often depends on daily self‐management, treatment adherence, glycemic target setting, and ongoing risk management. Although previous studies have described barriers to diabetes management in older adults and challenges in dementia care, these conditions have often been examined separately. Less is known about how physicians experience the combined disruption that occurs when dementia develops in patients already receiving diabetes care. In such situations, physicians must reconsider treatment goals, assess the feasibility of self‐management, involve family members or caregivers, and balance respect for patient autonomy with concerns about safety. Uncertainty may also arise regarding when and how to diagnose or disclose dementia, how strictly to pursue glycemic control, and how to coordinate care across professional boundaries.

The coexistence of diabetes and dementia may be understood through the sensitizing concepts of moral distress and role conflict. Moral distress may occur when physicians recognize what might be clinically or ethically desirable but are unable to achieve it because of cognitive decline, family circumstances, limited support systems, or uncertainty about prognosis [23]. Role conflict may also emerge when physicians are expected to reconcile disease‐oriented responsibilities with broader community‐based roles, such as supporting patients' life contexts, collaborating with other professionals, and coordinating care across medical and social domains [24]. These concepts are particularly relevant in Japan, where older adults with diabetes may be cared for by both primary care physicians and disease‐specific specialists, and where specialists may also function as de facto primary care providers in the absence of a strict gatekeeping system. In addition, Japan's dementia policy and community‐based integrated care initiatives emphasize linking medical care with long‐term care, caregiver support, and community resources [25]. Dementia diagnosis and disclosure may also be connected to access to long‐term care services, adding complexity to physicians' decision‐making and family involvement [26]. Existing qualitative studies from other healthcare systems provide valuable insights into dementia care and chronic disease management, but they do not fully explain how physicians in Japan's mixed‐care delivery context conceptualize and manage the ethical, relational, and practical tensions arising from diabetes–dementia comorbidity.

Against this background, this study aimed to explore how primary care physicians and diabetes specialists in Japan perceive and conceptualize difficulties in caring for older adults with type 2 diabetes and coexisting dementia. Rather than evaluating specific interventions or practice strategies, we focused on physicians' experiences of treatment‐goal adjustment, patient autonomy and safety, family involvement, care coordination, and emotional responses, as well as the ways in which they adapted their care within everyday clinical practice. Moral distress and role conflict were used as sensitizing concepts to frame the research question, while the analysis itself was conducted inductively using the SCAT method.

## 2 Methods

### 2.1 Study Design and Participants

This qualitative study was conducted in Japan using criterion‐based purposive sampling. We sought to recruit physicians who could provide rich accounts of caring for older adults with type 2 diabetes and coexisting dementia. Two physician groups were selected: primary care physicians certified as family medicine specialists under the Japanese board certification system and diabetes specialists accredited by the Japan Diabetes Society. This dual‐group design was chosen because, in Japan's healthcare system, both primary care physicians and disease‐specific specialists are involved in chronic disease management, and specialists may also function as de facto primary care providers in the absence of a strict gatekeeping system.

Eligibility criteria were as follows: board certification in either family medicine or diabetology; current or recent experience as an attending physician for older adults with type 2 diabetes, including patients with comorbid dementia; and sufficient clinical experience to reflect on difficulties in diabetes–dementia care. In practice, all included participants had at least 5 years of post‐certification clinical experience. “Extensive experience” was therefore operationalized not only by years of practice but also by direct clinical responsibility for this patient population. We aimed to include variation in specialty and practice setting, including community clinics, municipal hospitals, university hospitals, and affiliated outpatient clinics, to capture differences in clinical roles and care contexts rather than to achieve statistical representativeness.

Participants were identified through the professional networks of the research team. Potential participants were approached because they met the eligibility criteria and were expected to have substantial experience with the target patient population. The invitation included an explanation of the study purpose, the voluntary nature of participation, and confidentiality procedures. Some participants were professionally acquainted with members of the research team through regional clinical, educational, or academic activities; however, there were no direct supervisory or evaluative relationships between the interviewers and participants. Participants were informed that declining participation would have no disadvantage, and informed consent was obtained only after they had received written and verbal explanations of the study and had the opportunity to ask questions.

Initial eligibility was assessed based on physicians' specialty certification, years of clinical experience, and self‐reported experience caring for older adults with type 2 diabetes, including patients with comorbid dementia. Final inclusion in the analysis required that the interview contain a concrete clinical account of diabetes–dementia comorbidity. Fourteen physicians were invited to participate. Twelve physicians were included in the final analysis: six primary care physicians and six diabetes specialists. Two physicians were not included because, although they met the initial professional criteria, the cases discussed during early data collection focused primarily on dementia care and did not provide sufficient information about diabetes management in the context of comorbid dementia. No physician who met the final eligibility criteria and proceeded to the interview stage declined participation.

### 2.2 Demographics and Practice Setting

The participants included both male and female physicians aged between 30 and 50 years. The primary care physicians worked in diverse settings such as community clinics, regional hospitals, and university‐affiliated practices. Diabetes specialists were primarily based in university hospitals or large regional medical centers but often practiced in affiliated local institutions one to three times per week. Years of clinical experience post‐specialty certification ranged from 5 to 25 years.

### 2.3 Data Collection

Semi‐structured, in‐depth interviews were conducted by three members of the research team (S.T., D.S., and N.S.) either in person or via secure online platforms, typically in the evening. Each interview lasted approximately 60 min. All interviewers had prior experience in qualitative interviewing and were familiar with the study aims and interview guide before data collection began.

The interview guide was designed to anchor the discussion in the care of older adults with type 2 diabetes and coexisting dementia. At the beginning of each interview, participants were asked to recall one or more concrete cases in which an older patient with type 2 diabetes developed or had comorbid dementia. Follow‐up questions explored how dementia affected diabetes self‐management, treatment‐goal adjustment, medication or insulin use, family involvement, communication with patients and caregivers, interprofessional coordination, and physicians' emotional responses. When participants discussed dementia care in general terms, interviewers redirected the conversation to the diabetes–dementia context by asking how cognitive impairment influenced diabetes management and related decision‐making.

Most interviews were conducted by two interviewers. In these paired interviews, one interviewer primarily led the conversation while the other monitored coverage of the interview guide, asked supplementary questions, and took field notes. To minimize interviewer variability, the research team held preparatory meetings before data collection to confirm the purpose of each interview question, the intended use of probes, and procedures for redirecting the discussion to diabetes–dementia comorbidity when necessary. During the data collection period, the interviewers also discussed emerging issues after interviews and checked whether subsequent interviews consistently covered the core domains of the guide. The same core questions were used across all interviews to support comparability between the two physician groups.

The interview guide was refined after the first two preliminary interviews. The refinement did not change the core research focus or the main domains of inquiry. Rather, it clarified prompts so that participants would provide concrete accounts of diabetes–dementia comorbidity rather than discussing dementia care in general. Specifically, additional probes were added regarding how cognitive impairment affected diabetes self‐management, treatment‐goal adjustment, family involvement, and care coordination. Because these refinements were limited to clarifying probes and because the same core domains were retained, the research team judged that comparisons between primary care physicians and diabetes specialists remained appropriate.

All interviews were audio‐recorded with participants' permission and transcribed verbatim in Japanese. Transcripts were checked against the audio recordings for accuracy and were anonymized by removing personal names, institutional identifiers, and other potentially identifying workplace details before analysis. The interviews were conducted and analyzed in Japanese. Representative quotations selected for publication were translated into English by the research team and checked by bilingual members for accuracy and consistency with the original meaning. Transcripts were not returned to participants for member checking; instead, credibility was supported through interviewer debriefing, team‐based review of transcripts and coded segments, and discussion among researchers with different clinical and disciplinary backgrounds.

### 2.4 Data Analysis

Interview transcripts were analyzed using the Steps for Coding and Theorization (SCAT) method [27, 28]. SCAT is a qualitative analysis method suitable for relatively small‐scale interview data and involves a structured process of moving from textual data to concepts, storylines, and theoretical descriptions. In this study, analysis proceeded through the following steps. First, meaningful segments related to physicians' perceived difficulties in diabetes–dementia care were extracted from each transcript. Second, the researchers identified noteworthy words or phrases within each segment. Third, these phrases were paraphrased using words outside the original text to clarify their meaning. Fourth, concepts and explanatory phrases were generated to capture the underlying meaning of each segment. These concepts were then synthesized into storylines for each participant and subsequently compared across participants to develop themes and higher level typologies. To enhance transparency, examples of the coding process from several representative text clusters to conceptualization are provided in Table S1.

Two researchers (S.T. and D.S.) conducted the main analysis. To enhance analytic consistency, four transcripts, comprising two interviews with primary care physicians and two with diabetes specialists, were selected for independent coding by both researchers. These transcripts were selected using a stratified approach to ensure that both physician groups were represented. The two coders independently applied the SCAT process to these transcripts and then compared coding decisions, conceptual labels, and emerging interpretations. Differences were resolved through discussion, and when necessary, a third researcher was consulted. After the coding approach and preliminary interpretive framework had been agreed upon, the remaining transcripts were coded by the first coder and reviewed by the second coder, with particular attention to consistency of coding, fit between data extracts and themes, and differences between the two physician groups.

NVivo software was used to support data management rather than to substitute for interpretive analysis. Specifically, NVivo was used to organize transcripts, store coded segments, retrieve data extracts by theme and participant group, manage analytic memos, and compare themes across primary care physicians and diabetes specialists. Memos were used to record emerging interpretations, questions about coding boundaries, and decisions made during team discussions. The research team repeatedly reviewed coded segments and representative quotations to ensure that themes were grounded in the interview data and were not merely lists of abstract categories.

The analysis was conducted inductively, while moral distress and role conflict/role ambiguity were used as sensitizing concepts to frame the research question and support interpretation. These concepts were not used as fixed a priori coding categories. Instead, they helped the research team consider how physicians described tensions between standard disease management, patient autonomy, family involvement, interprofessional care, and their own emotional responses.

During the final stage of analysis, themes were compared within and across the two physician groups to identify shared and group‐specific patterns. Rather than treating the number of codes as indicating prevalence or importance, we interpreted themes in relation to the content, context, and depth of participants' narratives. Higher‐level typologies were developed by examining how individual themes clustered around broader dimensions of difficulty, including disruption of diabetes self‐management, negotiation of autonomy and safety, family and interprofessional involvement, treatment‐goal adjustment, and physicians' emotional responses.

### 2.5 Ethical Considerations

This study was approved by the Ethics Committee of Hino Municipal Hospital (Approval No. 2020–10). All participants received written and verbal explanations of the study purpose, voluntary nature of participation, confidentiality procedures, and their right to withdraw before providing informed consent.

All audio recordings, transcripts, consent forms, and analytic files were stored securely, and access was restricted to members of the research team. Consent forms and interview data were stored separately. Audio recordings and transcripts were assigned study identification numbers, and personal identifiers were removed during transcription and data processing.

Particular care was taken to protect participant anonymity because the study was conducted in a mid‐sized regional context where institutions, municipalities, or professional roles could potentially be recognizable. Names of individuals, institutions, municipalities, and specific workplace details were removed or generalized during transcription and reporting. Quotations selected for publication were reviewed to ensure that they did not contain details that could identify participants, patients, families, or healthcare institutions. Participant characteristics were reported in aggregated or broad categories where appropriate to reduce the risk of deductive disclosure.

## 3 Results

### 3.1 Participant Characteristics

This study was conducted in a regional healthcare context in the Chugoku region of Japan, where community clinics, municipal hospitals, university hospitals, and affiliated outpatient clinics coexist within the public health insurance system. The study area included both urban and rural catchment areas, and older adults with type 2 diabetes and comorbid dementia were often cared for through overlapping medical and long‐term care pathways involving physicians, family members, care managers, visiting nurses, and other community‐based professionals.

Twelve physicians participated: six primary care physicians and six diabetes specialists (Table 1). All participants treated older adults with type 2 diabetes, including patients with comorbid dementia. Primary care physicians tended to work in community‐based clinics or municipal hospitals and were often involved in longitudinal outpatient care and coordination with long‐term care professionals, whereas diabetes specialists were mainly based in university hospitals or larger regional hospitals and often provided specialist outpatient care with some inpatient or consultation responsibilities. These contextual differences were considered when interpreting the findings.

**TABLE 1:** Participant profiles.

Participant	Age	Gender	Specialty	Practice setting	Years in practice	Clinic volume	Urban/Rural practice	Outpatient/Inpatient emphasis
Dr. A	30's	Female	Family medicine	University Hospital	12	Moderate	Urban	Mixed outpatient‐inpatient
Dr. B	30's	Male	Family medicine	University Hospital	9	Moderate	Urban	Mixed outpatient‐inpatient
Dr. C	40's	Male	Family medicine	Municipal Hospital	12	Moderate	Mixed urban–rural	Mixed outpatient‐inpatient
Dr. D	40's	Male	Family medicine	Clinic	14	Low to moderate	Rural	Outpatient mainly
Dr. E	40's	Male	Family medicine	Municipal Hospital	15	Moderate	Mixed urban–rural	Mixed outpatient‐inpatient
Dr. F	30's	Female	Family medicine	Clinic	12	Low to moderate	Rural	Outpatient mainly
Dr. G	40's	Male	Diabetology	University Hospital	14	High	Urban/regional referral	Outpatient mainly with inpatient consultation
Dr. H	30's	Female	Diabetology	University Hospital	8	High	Urban/regional referral	Outpatient mainly with inpatient consultation
Dr. I	30's	Male	Diabetology	University Hospital	13	High	Urban/regional referral	Outpatient mainly with inpatient consultation
Dr. J	40's	Female	Diabetology	University Hospital	13	High	Urban/regional referral	Outpatient mainly with inpatient consultation
Dr. K	30's	Male	Diabetology	University Hospital	13	High	Urban/regional referral	Outpatient mainly with inpatient consultation
Dr. L	40's	Male	Diabetology	Municipal Hospital	21	Moderate to high	Mixed urban–rural	Mixed outpatient‐inpatient

*Note:* Clinic volume, urban/rural practice, and outpatient/inpatient emphasis were broadly categorized based on participants' main practice settings and interview data. These variables were used to describe practice context rather than to provide precise quantitative measures of patient volume or case mix.

### 3.2 Overview of Physician‐Reported Challenges

Themes and illustrative codes were developed separately for primary care physicians and diabetes specialists (Tables 2 and 3). The number of codes and themes is reported only to describe the analytic process and should not be interpreted as indicating the prevalence, intensity, or relative importance of difficulties in either physician group. The tables present thematic domains, operational definitions, and illustrative codes to clarify the conceptual boundaries among themes.

**TABLE 2:** Themes and illustrative codes in primary care physicians' experiences of diabetes–dementia care.

Themes	Operational definition	Illustrative codes
Discrepancy between ideal and real‐world care	Tensions between guideline‐based or professionally desirable diabetes care and what was realistically feasible in everyday clinical practice for patients with dementia	Struggling with the gap between standard protocols and real‐life practice
		Ethical dilemma between patient autonomy and clinical safety
		Frustration with unresolved clinical problems
Dementia‐specific challenges	Difficulties directly arising from cognitive decline, BPSD, impaired understanding, or altered communication in the context of diabetes management	Fear of losing patients' self‐control due to cognitive decline
		Diminished understanding of illness and poor self‐management
		Difficulties in communication and relationship‐building due to dementia
Patient‐centeredness	Efforts to tailor diabetes care to the patient's values, preferences, life circumstances, and capacity for shared decision‐making	Emphasis on individualized, context‐sensitive care
		Respect for patients' values and wishes
		Commitment to continuity and relational care
		Challenges in shared decision‐making
		Focus on the patient's environment and life goals
Respect for patient dignity	Ethical concern for preserving the patient's personhood, human rights, and life narrative, especially when cognitive decline limited autonomous expression	Attention to human rights and dignity
		Respect for patients' end‐of‐life views
Family systems in care	Recognition of family members as participants in diabetes–dementia care, including their roles as supporters, decision‐making partners, and sources of relational complexity	Providing lifestyle guidance involving family
		Recognizing family members as key supporters
Creative treatment strategies	Practical and relational adaptations used to make diabetes care safer, more feasible, and more acceptable in the patient's everyday life	Balancing safety and practicality in treatment goals
		Seeking realistic compromises
		“Tuning” care to suit the patient's circumstances
		Maintaining presence and empathy (“being beside”)
		Reflective learning from clinical experience
Interprofessional collaboration	Coordination of diabetes–dementia care with other professionals to share goals, distribute responsibility, and overcome complex care situations	Sharing care goals within the team
		Collaborating with other professionals
		Establishing egalitarian relationships beyond hierarchies
		Overcoming difficult situations through teamwork
Emotional aspects of physician experience	Physicians' affective and reflective responses to uncertainty, relational change, inability to achieve ideal care, and limits of professional control	Empathy for the patient's perspective
		Self‐blame due to limitations in care
		Uncertainty when making decisions without clear evidence
		Sadness from deteriorating relationships due to memory loss
		Difficulties in letting go of control
		Burden of keeping medical knowledge up to date
Paradigm conflicts in medical practice	Tensions between disease‐oriented biomedical care and broader family medicine perspectives that emphasize life context, relationships, and whole‐person care	Tensions between family medicine perspectives and biomedical models
		Shifting from a disease‐based model to a life‐contextual care model

**TABLE 3:** Themes and illustrative codes in diabetes specialists' experiences of diabetes–dementia care.

Themes	Operational definition	Illustrative codes
Discrepancy between ideal and real‐world care	Tensions between guideline‐based diabetes care or professionally desirable management and the complexity of real‐world care for patients with dementia	Tension between standard care and complex real‐life cases
		Anxiety about uncertainty related to patients and disease progression
		Feelings of futility when problem‐solving fails
		Self‐reflection on professional failures
Diabetes‐specific challenges	Difficulties specific to diabetes management, including asymptomatic disease progression, prognostic uncertainty, and patient factors that affect self‐management	Difficulty predicting outcomes due to asymptomatic progression
		Challenges arising from patients' psychological traits
Dementia‐specific challenges	Difficulties arising from cognitive decline, impaired understanding, altered decision‐making capacity, or dementia diagnosis and disclosure in the context of diabetes care	Difficulties in patient understanding and self‐management
		Challenges in providing lifestyle guidance
		Complexities in balancing patient autonomy and family involvement
		Difficulties in diagnosing and disclosing dementia
Patient‐centeredness	Efforts to understand and respond to the patient's perspective, context, narrative, and ongoing relationship, even within specialist diabetes care	Recognition of gaps between medical and patient perspectives
		Attention to individual patient contexts
		Supportive care tailored to unique patient and family situations
		Meaning found in attending to patient narratives
		Commitment to ongoing relationships
Family systems in care	Recognition of family members as essential participants in diabetes–dementia care, including their support roles and the psychological dilemmas created by family needs	Psychological dilemmas when considering family needs
		Necessity of involving family in care
Creative treatment strategies	Practical adaptations used to maintain safety and feasibility in diabetes care when standard treatment goals or self‐management expectations were difficult to sustain	Providing minimum‐safe‐level care in real‐world conditions
		Developing self‐devised strategies
		Reflective learning through experience
		Joy from small improvements
		Motivation drawn from patient/family gratitude
Emotional aspects of physician experience	Physicians' affective responses to uncertainty, patient vulnerability, relational closeness, and the emotional demands of managing diabetes–dementia comorbidity	Emotional coping as a professional
		Empathy with the patient's experience
		Feelings of compassion and closeness
Professional meaning despite difficulty	Positive meaning, fulfillment, or professional motivation derived from caring for patients and families despite clinical complexity and uncertainty	Gaining fulfillment through care despite challenges
		Discovering meaning in clinical practice

### 3.3 Higher‐Level Structure of Physician‐Reported Challenges

Taken together, the themes shown in Tables 2 and 3 suggest that dementia disrupted several assumptions underlying diabetes management, including patient self‐management, treatment adherence, glycemic target setting, and autonomous decision‐making. Both physician groups experienced clinical, ethical, relational, and emotional difficulties arising from this disruption, while also developing adaptations to make care safer and more feasible in everyday practice. However, they tended to emphasize different aspects of the care process according to their clinical roles and practice contexts. The following sections first describe themes shared by both groups and then present themes that were more prominent in each physician group.

### 3.4 Common Themes Shared by Both Physician Groups

**[Discrepancy Between Ideal and Real‐World Care]**

Both groups described a sense of dissonance between guideline‐based diabetes care and what was feasible in everyday practice when cognitive impairment affected self‐management. Rather than simply abandoning glycemic control, physicians repeatedly described having to reinterpret treatment goals in light of safety, feasibility, and the patient's living situation.

One diabetes specialist explained how relaxed glycemic targets for older adults with dementia provided some relief, while also acknowledging that even these goals could be difficult to achieve:

The guideline now allows more relaxed HbA1c targets for patients with dementia, and that makes me feel much more at ease. If we still had to aim for below 7% for everyone, that would be extremely difficult. For this patient, that would be completely unrealistic.

A primary care physician similarly described ambivalence after reducing medication intensity in accordance with guideline‐based reasoning:

Even though I know that, according to the guideline, we do not need to control glucose so strictly in someone with dementia, when the numbers get worse I still wonder what the next step should be, and whether what I did was really the right approach.

**[Dementia‐Specific Challenges]**

Participants described cognitive decline not only as a diagnostic issue but as a practical disruption to diabetes care. Forgetfulness, impaired understanding, unpredictable eating behavior, and difficulty learning new routines made conventional lifestyle guidance and medication management difficult.

One diabetes specialist described the difficulty of providing lifestyle guidance when the patient could not remember the behavior that had worsened glycemic control:

I want to improve the patient's glucose levels, but the patient is not doing something bad on purpose and really does not remember doing it. So it is difficult to give guidance directly to the patient, and I end up placing more emphasis on talking with the family.

Another specialist emphasized that even treatment changes that seemed simple to physicians could be difficult for patients with cognitive impairment:

A treatment that I thought was simple was still difficult and complicated for the patient. I realized that starting something new, or learning a new routine, is itself difficult for someone with dementia.

**[Patient‐Centeredness]**

Both primary care physicians and diabetes specialists emphasized the importance of respecting patients' values, preferences, and understanding of illness. However, they also noted the difficulty of maintaining patient autonomy when cognitive function declined or fluctuated.

One primary care physician described beginning the relationship by asking how the patient understood their illness and medication:

When I first take over a patient, I try to ask about their life history and what they think about their medications. I also ask, ‘What illness are you coming to the clinic for?’ Some patients understand their condition, while others simply say, ‘I just take the medicine the previous doctor prescribed’. So I start from there, by explaining and gradually understanding their values.

A diabetes specialist similarly described trying to preserve the patient's participation in decision‐making, even when memory impairment made sustained understanding difficult:

I try to explain things in simple words so that the patient can understand at that moment. I do not think we can proceed with treatment unless the patient is at least momentarily convinced. At the same time, I discuss the longer‐term treatment plan with the wife.

**[Family Systems in Care]**

Family members and caregivers were described as essential sources of information and support, but participants also noted that family involvement could be limited, emotionally complex, or burdensome. Physicians therefore had to negotiate not only patient care but also the capacity and circumstances of family caregivers.

One diabetes specialist described how family information was necessary to understand unexpected deterioration in glycemic control:

When the numbers suddenly got worse, I suspected that the patient must be eating something, but I could not know what it was from the patient alone. It was only by asking the family carefully that we began to understand what was happening.

Another specialist noted that family support could not be assumed without considering caregiver burden:

The wife was supporting him very well, but I also felt that we had to be careful not to increase her burden too much.

**[Creative Treatment Strategies]**

In response to these difficulties, physicians described practical adaptations rather than standardized solutions. These included relaxing targets, simplifying medications, avoiding hypoglycemia, using family or facility staff as information sources, and seeking a “minimum safe level” of care.

One diabetes specialist described adapting treatment to reduce the burden of medication management:

We tried switching to a once‐weekly medication so that, even with fewer opportunities for medication supervision, the patient could take it more reliably.

A primary care physician described the difficulty of explaining such adjustments to facility staff when changes in medication led to fluctuations in glucose levels:

The previous doctor had kept the same medication and the numbers looked stable, but after I changed the treatment, it seemed as if things were changing again and again. I wondered whether the people around the patient saw my approach as inconsistent.

**[Emotional Aspects of Physician Experience]**

Both groups reported emotional burdens, such as frustration, helplessness, and guilt, particularly when ideal standards of care were unattainable. The emotional toll of navigating uncertain outcomes and ethical ambiguity was a common experience.

One primary care physician described the frustration of having no clearly correct option:

I knew this situation wasn't good, but there wasn't any decisive solution. The patient needed insulin—there was no way around that. But when insulin itself became the problem, I honestly felt stuck.

A diabetes specialist described the challenge of adjusting to unpredictable behavior in patients with undiagnosed dementia:

If I know it's dementia, I can accept it—like, ‘Okay, that's just the dementia talking’. But when I don't know, and the patient says or does something strange, I get irritated at first. It takes time to understand the person behind those actions.

### 3.5 Themes More Prominent Among Primary Care Physicians

**[Respect for Patient Dignity]**

Primary care physicians emphasized maintaining patient dignity and identity, even as cognitive impairment progressed. They prioritized a relational, person‐centered approach and avoided overly paternalistic care models. One physician recounted a moment of ethical reflection when a patient passively accepted insulin:

The patient said, ‘If you think I should take insulin, go ahead, I don't mind’. But that made me pause. Thinking back to how he was a year or two ago, I wondered—would this really have been the life he wanted? It wasn't exactly advance care planning, but I questioned whether this path honored his vision for his life.

**[Interprofessional Collaboration]**

Primary care physicians frequently collaborated with visiting nurses, care managers, and social workers. While this team‐based approach was seen as essential, systemic barriers and unequal hierarchies occasionally limited its effectiveness.

**[Paradigm Conflicts in Medical Practice]**

Some primary care physicians described tension between disease‐oriented diabetes management and the broader responsibilities of generalist care. One physician explained this as a conflict between referral and continued responsibility:

As long as I am practicing general medicine, I feel I should not refer too easily. I want to think about whether there is something more I can do myself. But at the same time, I still wonder what the right approach is.

### 3.6 Themes More Prominent Among Diabetes Specialists

**[Diabetes‐Specific Challenges]**

Specialists noted that the asymptomatic nature of diabetes and variability in prognosis made disease monitoring and treatment adjustments particularly difficult when cognitive decline was present. They also mentioned that certain personality traits in patients hindered self‐management.

**[Professional Meaning Despite Difficulty]**

Despite these difficulties, some diabetes specialists described moments of fulfillment when treatment adjustments improved the patient's condition or when patients and families expressed appreciation. One specialist recalled:

When a patient says, ‘My blood sugar levels have been bad for so long, but thanks to your adjustments, they've finally improved’, that really means a lot. And I'll say, ‘No, you did the work—I just made suggestions’. That kind of exchange gives me a real sense of purpose.

Another specialist described family appreciation as particularly meaningful in dementia care:

For patients with dementia, it is often the family rather than the patient who says thank you. Families are really struggling, so when they express gratitude, I feel that the care has meaning.

## 4 Discussion

This qualitative study explored the professional difficulties faced by primary care physicians and diabetes specialists in Japan when managing older patients with type 2 diabetes and coexisting dementia. While both groups reported shared challenges—such as balancing patient‐centeredness with safety concerns, navigating behavioral symptoms of dementia, and managing emotional burden—differences in emphasis emerged in how these challenges were conceptualized and addressed. Primary care physicians emphasized dignity, interprofessional collaboration, and life‐contextual care, whereas diabetes specialists tended to focus on disease‐specific uncertainties, emotional coping, and pragmatic treatment compromises.

The differences observed between primary care physicians and diabetes specialists should be interpreted cautiously. Rather than demonstrating the direct influence of educational background or training orientation, our findings suggest that physicians' narratives varied according to their clinical roles, practice settings, care responsibilities, and professional backgrounds. Primary care physicians more often described difficulties in terms of dignity, life context, longitudinal relationships, and interprofessional coordination, whereas diabetes specialists more often emphasized disease‐specific uncertainty, treatment‐goal adjustment, dementia diagnosis and disclosure, and pragmatic adaptations to maintain safety. These differences may be related to professional training, but the present study was not designed to determine causal explanations.

These findings nevertheless point to possible areas for future educational and organizational support. Physicians involved in diabetes–dementia care may benefit from opportunities to reflect on treatment‐goal adjustment, patient autonomy and safety, family involvement, ethical uncertainty, and interprofessional coordination. However, the present study did not directly examine institutional hierarchy, consultation time, educational content, or the functioning of diabetes care teams. Therefore, we avoid interpreting the less prominent discussion of interprofessional collaboration among diabetes specialists as evidence of a more individual or protocol‐driven professional culture. Future studies should examine how specific educational experiences, professional roles, organizational factors, consultation structures, and team‐based care arrangements influence physicians' approaches to diabetes–dementia care.

Given Japan's rapidly aging society and the increasing coexistence of diabetes and dementia, our findings suggest that physicians may need support in negotiating treatment‐goal adjustment, family involvement, ethical uncertainty, and interprofessional coordination. However, because this study was based on physician interviews only, it cannot determine which system‐level interventions would be most effective. Future research should include patients, family caregivers, allied healthcare professionals, administrators, and policymakers to examine how shared care planning and interprofessional coordination can be supported in everyday diabetes–dementia care.

Our findings also suggest the need to reconsider chronic disease care when cognitive decline undermines self‐management, treatment adherence, and conventional shared decision‐making. In diabetes–dementia care, physicians must navigate a gray zone between respecting autonomy and ensuring safety, while also considering family circumstances and the patient's quality of life. These situations require not only technical adjustment of glycemic targets but also ethical and relational judgment.

Our findings both align with and extend previous studies from other healthcare systems. Similar to reports from the UK and US, participants in this study described difficulties in aligning dementia care with chronic disease management, particularly when cognitive decline undermined self‐management, treatment adherence, and shared decision‐making. Prior studies have also identified barriers such as fragmented care coordination, limited dementia‐related support, time constraints, and the challenge of involving families in care [20, 29]. Likewise, the importance of relational continuity and team‐based care has been emphasized in primary care literature [30, 31]. In this respect, our findings suggest that diabetes–dementia comorbidity creates dilemmas that are shared across aging healthcare systems.

At the same time, the Japanese context adds a distinctive dimension to these challenges. In Japan's non‐gatekeeping healthcare system, diabetes specialists may continue to provide longitudinal outpatient care and may function partly as de facto primary care providers for older adults with diabetes. In addition, diabetes–dementia care often intersects with long‐term care pathways involving family caregivers, care managers, visiting nurses, and community‐based services. Thus, the challenge in Japan is not only the fragmentation of dementia and chronic disease care but also the negotiation of shared responsibility between primary care physicians, disease‐specific specialists, families, and long‐term care professionals.

This study has several limitations, many of which are inherent to its qualitative design. First, the sample was small and geographically limited to one region in Japan. Although the sample included both primary care physicians and diabetes specialists working in different practice contexts, it may not capture the full diversity of institutional, regional, and cultural perspectives on diabetes–dementia care.

Second, participants were recruited through the professional networks of the research team, and some participants were professionally acquainted with the interviewers through regional clinical, educational, or academic activities. Although participation was voluntary and confidentiality was emphasized, these preexisting professional relationships may have influenced participants' willingness to participate or the way they described their experiences. In addition, the researchers' own clinical backgrounds and interpretive roles may have shaped data collection and analysis, although reflexive discussion and team‐based review were used to reduce this influence.

Third, comparisons between primary care physicians and diabetes specialists should be interpreted cautiously. The two groups differed not only in specialty but also in practice settings, clinic volume, urban/rural practice context, and outpatient/inpatient emphasis. Therefore, differences in the themes emphasized by each group should not be interpreted as reflecting specialty alone. Rather, they may reflect the combined influence of professional role, care setting, patient population, and clinical responsibility.

Fourth, although we judged the data to be sufficient for the focused aim of this exploratory study, we did not use saturation as a simple numerical threshold. During data collection and analysis, the research team considered that later interviews were no longer generating major new thematic domains and that the sample provided adequate information power because the research question was focused and the participant groups were relatively specific. Nevertheless, additional interviews in other regions or institutional contexts might have generated further variation.

Fifth, the study focused exclusively on physicians and did not include the perspectives of patients, family caregivers, care managers, nurses, allied healthcare professionals, administrators, or policymakers. As a result, the findings cannot fully explain how diabetes–dementia care is experienced by other stakeholders or how interprofessional and organizational arrangements function in everyday practice.

Finally, interviews were conducted and analyzed in Japanese, and selected quotations were translated into English for publication. Although translations were checked for accuracy and consistency with the original meaning, some nuance may have been lost in translation. The findings are also situated within Japan's healthcare and long‐term care systems, including the absence of a strict gatekeeping primary care system and the involvement of long‐term care professionals. Therefore, transferability to other healthcare systems should be considered carefully.

Further research should include multi‐stakeholder interviews and longitudinal ethnographic studies to explore how ethical, educational, and systemic factors intersect in real‐world care settings. Implementation studies are also needed to evaluate models that bridge family medicine and specialty care—particularly in managing multimorbidity in older adults.

## 5 Conclusions

This qualitative study showed that primary care physicians and diabetes specialists in Japan experience overlapping but differently emphasized challenges when caring for older adults with type 2 diabetes and coexisting dementia. Dementia disrupted key assumptions of diabetes care, including patient self‐management, treatment adherence, glycemic target setting, and autonomous decision‐making. Physicians therefore had to negotiate treatment goals, patient autonomy and safety, family involvement, interprofessional coordination, and emotional uncertainty in everyday practice.

Differences between primary care physicians and diabetes specialists should be interpreted as variations in emphasis shaped by clinical roles, practice settings, and care responsibilities, rather than as fixed or opposing professional orientations. Primary care physicians more often emphasized dignity, life context, continuity, and interprofessional coordination, whereas diabetes specialists more often emphasized disease‐specific uncertainty, treatment‐goal adjustment, dementia diagnosis and disclosure, and pragmatic adaptations to maintain safety.

These findings suggest several possible areas for future educational and organizational support. Training for physicians involved in diabetes–dementia care could focus on individualized glycemic target setting, communication with patients and family caregivers, assessment of decision‐making capacity, ethical reflection on autonomy and safety, and collaboration with long‐term care professionals. Such training may be relevant for both primary care physicians and disease‐specific specialists, particularly those who provide longitudinal care for older adults with multimorbidity. Future studies should evaluate the feasibility and outcomes of these educational and organizational approaches, including their effects on physicians' confidence, interprofessional collaboration, patient‐centered decision‐making, and perceived burden.

## Author Contributions

**Toshihiro Hamada:** conceptualization, methodology, validation, writing – review and editing. **Shin‐ichi Taniguchi:** conceptualization, methodology, software, investigation, validation, formal analysis, visualization, project administration, writing – original draft, writing – review and editing, data curation. **Naomi Sunami:** conceptualization, methodology, validation, writing – review and editing, supervision. **Yuma Otsuka:** conceptualization, methodology, validation, writing – review and editing. **Daisuke Son:** conceptualization, methodology, software, investigation, validation, formal analysis, supervision, visualization, project administration, writing – review and editing. **Kazuoki Inoue:** conceptualization, methodology, validation, writing – review and editing. **Shohei Taniguchi:** conceptualization, methodology, validation, writing – review and editing. **Shintaro Imaoka:** conceptualization, methodology, writing – review and editing, validation. **Tsubasa Nakai:** conceptualization, methodology, validation, writing – review and editing.

## Funding

This work was supported by the Japan Society for the Promotion of Science (JSPS) KAKENHI [Grant‐in‐Aid for Scientific Research (C), Grant Number 19K10484, 2019–2023], for the project titled “The association between illness recognition process and healthcare barrier in elderly residents with diabetes.”

## Conflicts of Interest

The authors declare no conflicts of interest.

## Supporting information


**Table S1:** Examples of the SCAT coding process.

## Data Availability

The data that support the findings of this study are available from the corresponding author upon reasonable request.
