# Supplementary material for: Medical Challenges in Caring for Older Adults With Type 2 Diabetes and Dementia: A Qualitative Interview Study Using SCAT to Compare Primary Care Physicians and Diabetes Specialists in Japan
Source: J Gen Fam Med. 2026 Jul 26;27(4):e70154. doi: 10.1002/jgf2.70154 (PMC13402852; doi:10.1002/jgf2.70154)
Supplement: Supplementary file 1 — Table S1: Examples of the SCAT coding process. [file JGF2-27-e70154-s001.docx]

**Supplementary Table 1. Examples of the SCAT coding process**

| **Text cluster / transcript excerpt** | **Noteworthy words or phrases from the text** | **Paraphrased words or phrases outside the text** | **Concepts generated from the excerpt** | **Storyline / theoretical description** | **Related theme** |
| --- | --- | --- | --- | --- | --- |
| “The guideline now allows more relaxed HbA1c targets for patients with dementia, and that makes me feel much more at ease. If we still had to aim for below 7% for everyone, that would be extremely difficult. For this patient, that would be completely unrealistic.” | relaxed HbA1c targets; dementia; feel more at ease; below 7%; unrealistic | adjustment of glycemic goals; relief from strict disease-control expectations; recognition of infeasible standard targets | Tension between guideline-based targets and feasible care; reinterpretation of diabetes control in dementia care; safety-oriented goal adjustment | The physician experienced relief when guidelines allowed more relaxed glycemic targets for older adults with dementia. At the same time, the excerpt shows that conventional glycemic control goals could become unrealistic when dementia disrupted self-management. The physician therefore had to reinterpret diabetes care from strict disease control toward feasible and safer treatment goals. | Discrepancy Between Ideal and Real-World Care / Creative Treatment Strategies |
| “I want to improve the patient’s glucose levels, but the patient is not doing something bad on purpose and really does not remember doing it. So it is difficult to give guidance directly to the patient, and I end up placing more emphasis on talking with the family.” | improve glucose levels; not on purpose; does not remember; difficult to give guidance; talking with the family | impaired intentionality; memory-related loss of self-management; limits of patient education; shift toward family-mediated care | Cognitive impairment as a disruption to lifestyle guidance; family involvement as a compensatory strategy; limits of conventional self-management support | The physician recognized that poor glycemic control was not simply a matter of non-adherence, because the patient could not remember the relevant behavior. This made direct lifestyle guidance difficult and shifted the focus of care toward communication with family members. | Dementia-Specific Challenges / Family Systems in Care |
| “When I first take over a patient, I try to ask about their life history and what they think about their medications. I also ask, ‘What illness are you coming to the clinic for?’ Some patients understand their condition, while others simply say, ‘I just take the medicine the previous doctor prescribed.’ So I start from there, by explaining and gradually understanding their values.” | life history; what they think about medications; what illness; understand their condition; gradually understanding values | eliciting illness understanding; exploring medication meaning; relational assessment; value-oriented communication | Patient-centered understanding of illness and treatment; gradual construction of shared decision-making; attention to life context | The physician began care by exploring the patient’s understanding of illness, medication, and life history. Rather than assuming that the patient understood the purpose of diabetes treatment, the physician built care gradually through explanation and attention to the patient’s values. | Patient-Centeredness |
| “I knew this situation wasn’t good, but there wasn’t any decisive solution. The patient needed insulin—there was no way around that. But when insulin itself became the problem, I honestly felt stuck.” | wasn’t good; no decisive solution; needed insulin; insulin became the problem; felt stuck | clinical impasse; unavoidable treatment risk; conflict between necessary therapy and practical safety; helplessness | Moral and clinical uncertainty; treatment dilemma caused by dementia-related care limitations; emotional burden of unresolved responsibility | The physician experienced a clinical impasse in which insulin was medically necessary but became difficult or unsafe because of the patient’s dementia-related circumstances. This created emotional burden and uncertainty, reflecting the difficulty of achieving ideal care under real-world constraints. | Emotional Aspects of Physician Experience / Discrepancy Between Ideal and Real-World Care |
| “The patient said, ‘If you think I should take insulin, go ahead, I don’t mind.’ But that made me pause. Thinking back to how he was a year or two ago, I wondered—would this really have been the life he wanted? It wasn’t exactly advance care planning, but I questioned whether this path honored his vision for his life.” | go ahead; I don’t mind; made me pause; life he wanted; honored his vision | passive acceptance; diminished expression of preference; retrospective consideration of values; dignity-oriented decision-making | Ethical concern for preserving personhood; uncertainty about authentic preference; dignity and life narrative in treatment decisions | The patient’s passive acceptance of insulin led the physician to question whether the treatment aligned with the patient’s earlier values and life narrative. This excerpt shows how diabetes treatment decisions became ethically complex when dementia limited the patient’s ability to express autonomous preferences. | Respect for Patient Dignity / Patient-Centeredness |

*Note.* The original interview transcripts were in Japanese. The transcript excerpts shown in this table were translated into English for publication. The SCAT analysis was conducted using the Japanese transcripts, and the English excerpts are provided here to illustrate the analytic process. The examples are illustrative and do not represent the full coding process.
